# Comparison of Different Protein Emulsifiers on Physicochemical Properties of β-Carotene-Loaded Nanoemulsion: Effect on Formation, Stability, and In Vitro Digestion

**DOI:** 10.3390/nano11010167

**Published:** 2021-01-11

**Authors:** Yanlong Liu, Chang Liu, Shenyi Zhang, Jishu Li, Huanyu Zheng, Hua Jin, Jing Xu

**Affiliations:** 1 College of Art and Science, Northeast Agricultural University, Harbin 150030, China; liuyanlonghyy@163.com (Y.L.); lovekursaal@163.com (C.L.); zsylq123@163.com (S.Z.); ljswy123@163.com (J.L.); 2 College of Food Science, Northeast Agricultural University, Harbin 150030, China; zhenghuanyu1@163.com; 3 Heilongjiang Green Food Science Research Institute, Harbin 150028, China; 4 National Research Center of Soybean Engineering and Technology, Harbin 150028, China

**Keywords:** protein, β-carotene, nanoemulsions, in vitro digestion, physicochemical stability

## Abstract

In this study, β-carotene-loaded nanoemulsions are emulsified using four biomacromolecular proteins—peanut protein isolate (PPI), soy protein isolate (SPI), rice bran protein isolate (RBPI), and whey protein isolate (WPI)—in order to explore their emulsion stability and in vitro digestion characteristics. All four nanoemulsions attained high encapsulation levels (over 90%). During the three-stage in vitro digestion model (including oral, gastric, and small intestine digestion phases), the PPI-emulsified nanoemulsion showed the highest lipolysis rates (117.39%) and bioaccessibility (37.39%) among the four nanoemulsions. Moreover, the PPI-emulsified nanoemulsion (with the smallest droplet size) also demonstrated the highest stability during storage and centrifugation, while those for the RBPI-emulsified nanoemulsion (with the largest droplet size) were the lowest. In addition, all four nanoemulsions showed superior oxidation stability when compared with the blank control of corn oil. The oxidation rates of the PPI- and WPI-stabilized groups were slower than the other two groups.

## 1. Introduction

In the food industry, nanoemulsion delivery systems are applied to encapsulate and protect lipophilic bioactive nutrients, such as vitamin E, omega3 fatty acids, and curcumin, due to their high stability and encapsulating effect [1,2,3]. For nanoemulsions, the selection of an emulsifier is always an essential topic [4,5]. Jo et al. [6] used the microfluidic method to stabilize nanoemulsions and found that the nanoemulsion emulsified by whey protein isolate (WPI) had better physicochemical stability than that by Tween 20. Minaxi et al. [7] prepared a clove oil nanoemulsion emulsified by sodium caseinate (5%) and pectin (0.1%), which had a particle size of 172.1 ± 4.39 nm, high encapsulation efficiency (88%), and high stability. The work of Hu et al. [8] indicated that all nanoemulsions emulsified by casein, WPI, and soy protein isolate (SPI) have certain antioxidant stability. In the research of Fan et al. [9], whey protein isolate–dextran conjugates (WPI–Dex) with different molecular weights were prepared and used as emulsifiers. Their experimental results showed that WPI–Dex of 5 kDa could significantly improve the nanoemulsion antioxidant capacity, where a 70 kDa nanoemulsion stabilized by WPI–Dex could significantly inhibit the degree of lipolysis and the bioaccessibility of β-carotene. Thus, it can be stated that nanoemulsions prepared with different emulsifiers often differ in terms of the stability and bioaccessibility of the encapsulated bioactive compounds. Therefore, it is necessary to compare various emulsifiers for nanoemulsion preparations.

In recent years, plant proteins, with the advantages of wide range of sources, low prices, and high nutritional values, have attracted the widespread interest of researchers. More and more researchers have focused on the emulsifying properties of plant proteins. Aoki et al. [10] found that the 7S subunit in soybean protein had better solubility and emulsifying properties than the 11S subunit under different pH conditions. Chen et al. [11] showed that limited enzyme modification or heat treatment could improve the emulsifying properties of peanut protein isolate. In the research of O’Sullivan et al. [12], ultrasound treatment (34 W/cm^2^) was used to modify peanut and pea protein isolates. Their experimental results showed that the ultrasonic treatment could reduce the molecular weight and interfacial tension of proteins, thus improving the protein emulsifying properties. The studies mentioned above illustrate that in terms of food safety, the use of biomacromolecular surfactants as emulsifiers has received much attention from researchers.

The digestion of loaded nanoemulsions by the human digestive system is a complex process. Digestive fluids such as glycoprotein, mucin, and pepsin can drastically change the structure and dispersion state of the emulsion. In addition, the metabolism of nanoemulsions is also affected by their composition and structure, including particle size, interfacial structure, stability, and material properties, among others [13]. Therefore, investigation of the nanoemulsion fate during in vitro digestion is useful in helping us analyze the release and bioaccessibility of nutrients. Liu et al. [14] used an in vitro digestion model to compare the mean particle size, ζ-potential, nanostructure, and bioaccessibility of β-carotene-loaded nanoemulsion emulsified by decaglycerol monolaurate (ML750), soluble soybean polysaccharide (SSPS), and whey protein isolate (WPI). The ML750-stabilized nanoemulsion had a smaller particle size and, so, a large specific surface area, thus producing faster contact with digestive fluids in the gastric environment. However, in the simulated intestine, the soluble fiber of SSPS may inhibit β-carotene micellization in the emulsion, leading to the lowest release rate of β-carotene, while the WPI-stabilized emulsion presented the highest release rate, thus indicating that the difference during nanoemulsion digestion was significant for different emulsifiers.

At present, there are many studies in the literature on the in vitro digestion of small molecules and animal protein nanoemulsions. However, scarce studies have been reported about lipophilic nutrient release or the digestion characteristics of nanoemulsions stabilized by plant proteins. Moreover, it is also necessary to compare the effects of plant and animal proteins on nanoemulsion stability and digestion. Accordingly, our research focused on the use of natural plant proteins—peanut protein isolate (PPI), soybean protein isolate (SPI), and rice bran protein isolate (RBPI)—as emulsifiers, compared to an animal protein—whey protein isolate (WPI)—as a control, in order to stabilize β-carotene-loaded nanoemulsions. The centrifugation stability, pH stability, storage stability, and oxidation stability of nanoemulsions were analyzed. Furthermore, an in vitro digestion model of nanoemulsions was explored. The performance of the nanoemulsions during in vitro digestion was evaluated by the mean particle size and ζ-potential, while the lipolysis rates and bioaccessibility of β-carotene were also discussed.

## 2. Materials and Methods

### 2.1. Materials

Defatted peanut powder and rice bran powder were purchased from Changshou Food Co. (Qingdao, China). Low-temperature defatted soybean meal was purchased from Harbin High-Tech Co. (Harbin, China). Whey protein isolate (WPI, >90%) was purchased from Zhengzhou Jiangda Biotechnology Co. (Zhengzhou, China). β-carotene was purchased from Biotoped Co. (Beijing, China). Pepsin was purchased from Solarbio (Beijing, China) with activity of 3000–3500 NFU/g. Porcine pancreatic enzyme was purchased from Sigma-Aldrich (St. Louis, MO, USA) with activity of 4xJP. Bile salt extract was purchased from Solarbio (Beijing, China). Butylated hydroxytoluene (BHT) was purchased from Merck Co. (Darmstadt, Germany). Corn oil was purchased from a local grocery store. Trichloroacetic acid (TCA) was purchased from Samchun Pure Chemicals Co. Ltd. (Seoul, Korea). Other reagents were all analytical grade.

### 2.2. Extraction of Isolated Proteins and Preparation of Nanoemulsions

The isolated proteins of peanut (PPI), soybean (SPI), and rice bran (RBPI) were extracted based on previous research [15,16,17]. Defatted peanut flour, soybean flour, rice bran flour, and ultrapure water were mixed in a ratio of 1:10 (*w/w*), the pH was adjusted to 9.5, 8.0, and 9.0 with 2.0 M NaOH, respectively, stirred at room temperature for 2 h, and then centrifuged at 3500× *g* for 15 min. After centrifugation, the supernatant was collected by filtration with three layers of gauze (80 mesh) and adjusted to pH 4.5 with 2.0 M HCl. The sample was allowed to stand overnight and centrifuged at 3500× *g* for 20 min to collect the protein precipitation in the pellet phase. After that, the protein concentration is determined by the biuret method. Based on our previous research, PPI, SPI, RBPI, and WPI were added at appropriate concentrations (2%, 3%, 2%, and 4% *w/v*, respectively) to the aqueous phase [18]. The protein purities of PPI, SPI, and RBPI were determined by the Kjeldahl method. In brief, the sample was weighed, dried at 105 °C, and put into a 50 mL Kjeldahl flask with 300 mg K_2_SO_4_–CuSO_4_ and 3 mL H_2_SO_4_. After the digestion and distillation in a fume hood, 0.05 M HCl was used to titrate until the boric acid indicator turned from green to purple.

(1) Sample total nitrogen (mg) = (A−B)×C×14×100/20

where A represents the volume of added HCl consumed during sample titration; B represents the volume of added HCl consumed during blank titration; C represents the concentration of HCl; 14 is the relative molecular weight of nitrogen; 20 represents the volume of diluted digestion solution used for distillation; and 100 represents the volume of diluted digestive fluid.

(2) $$\mathrm{The}\;\mathrm{purity}\;\mathrm{of}\;\mathrm{the}\;\mathrm{protein}\;\left(\%\right)=\frac{\;\mathrm{the}\;\mathrm{total}\;\mathrm{nitrogen}\;\mathrm{content}\;\mathrm{of}\;\mathrm{the}\;\mathrm{sample}\;\left(\mathrm{mg}\right)\;\times \;6.25}{\mathrm{the}\;\mathrm{total}\;\mathrm{mass}\;\mathrm{of}\;\mathrm{the}\;\mathrm{sample}\;\left(\mathrm{mg}\right)}$$

The resultant purities of PPI, SPI, and RBPI were 83%, 84%, and 81%, respectively [16].

For the oil phase, β-carotene was dissolved in corn oil at a concentration of 0.4% (*w/v*) with heating by ultrasound (55 °C, <5 min). Then, the solution was stirred at room temperature for about 1 h to ensure full dissolution. It should be noted that during the whole mixing process, light exposure should be avoided to prevent the decomposition of β-carotene. The aqueous phase (95% *v/v*) and oil phase (5% *v/v*) were homogenized using a homogenizer (FJ200-SH, Shanghai Sample Model Factory, Shanghai, China) at 10,000 rpm for 4 min to obtain a crude emulsion (pH = 7.0). The entire homogenization process was carried out at room temperature. Then, an ultrasonic processor with an ultrasonic probe (Ningbo Xinzhi Biotechnology Co., Ltd., Ningbo, China) was used with ultrasonic power at 500 W for 20 min under frequencies of 20 Hz. The temperature was controlled at 25 °C by an ice water bath to prevent the protein denaturation during the preparation.

### 2.3. Determination of Mean Particle Size and ζ-Potential

The mean particle size and polydispersity index (PDI) of the nanoemulsions were all volume-based, which was determined using dynamic light scattering (Zetasizer Nano-ZS90, Malvern Instruments, Worcestershire, UK). The ζ-potential of the nanoemulsion system was investigated using electrophoresis (Zetasizer Nano-ZS90, Malvern Instruments, Worcestershire, UK) [19,20]. In order to avoid multiple light scattering effects, the emulsion was diluted (1:100 *v/v*) with 10 mmol/L pH = 7.0 phosphate buffer solution (8 g NaCl, 0.2 g KCl, 1.44 g Na_2_HPO_4_, 0.24 g KH_2_PO_4_ per liter) and mixed thoroughly. The refractive indices of the continuous phase and the dispersed phase were set as 1.33 and 1.47, respectively. The mean particle size and ζ-potential of the nanoemulsions were measured at 25 °C [21].

### 2.4. Measurement of β-Carotene Content and Encapsulation Efficiency of β-Carotene Nanoemulsions

Determination of the β-carotene content in the nanoemulsion was referred to the method of Hou et al. with slight modification [22]. First, 1 mL of emulsions was diluted 10 times with distilled water. Then, the β-carotene was extracted with 1 mL of chloroform three or four times, until the sample was colorless. All the chloroform phase was pooled together and adjusted to appropriate concentration for detection. Absorbance was measured at 450 nm by the spectrophotometer (T6 New Century UV-Vis Spectrophotometer, Persee analytics, Beijing, China). The content of β-carotene was obtained by referring to the standard curve of β-carotene measured under the same conditions. In our experiment, the standard curve of β-carotene-chloroform was as follows:

(3) y = 0.0318x + 0.0004 (= 0.9997)

where x represents the concentration of β-carotene and y represents the absorbance at the corresponding concentration.

Encapsulated efficiency was calculated by the following equation:

(4) $$\mathrm{Encapsulated}\;\mathrm{efficiency}\left(\%\right)=\frac{\mathrm{Encapsulated}\;\beta -\mathrm{carotene}\left(\mathrm{mg}\right)}{\mathrm{total}\;\beta -\mathrm{carotene}\;\mathrm{input}\left(\mathrm{mg}\right)}\times 100\%.$$

### 2.5. In Vitro Digestion Model

A three-stage in vitro digestion model was used to simulate the digestion conditions [23].

#### 2.5.1. Initial Phase

The 4 mL PPI-, SPI-, RBPI-, and WPI-stabilized nanoemulsions were maintained in an incubating shaker and preheated to 37 °C.

#### 2.5.2. Oral Phase

Next, 4 mL of simulated saliva fluid, containing 0.03 g/mL mucin and various salts, was preheated to 37 °C and mixed to the same volume of the initial nanoemulsion [24]. After adjusting the pH to 6.8–7.0 by 0.1 M HCl, the samples were placed in an incubated shaker at 37 °C for 10 min with continuous stirring at 100 rpm in order to simulate oral digestion.

#### 2.5.3. Gastric Phase

Then, 10 mL of simulated gastric fluid (3.2 mg/mL pepsin and 0.15 M NaCl) was added to the digested mixture after the oral phase, the pH value was adjusted to 2.0 by 2.0 M HCl, and the 18 mL mixture was continuously stirred at 100 rpm at 37 °C for 1 h to simulate gastric digestion.

#### 2.5.4. Small Intestine Phase

Then, the pH of the digested mixture from the gastric phase was adjusted to 7.0 by 2.0 M NaOH. Thereafter, 15 mL of simulated intestinal fluid (1.0 mg/mL porcine pancreatic enzyme, 20.0 mg/mL bile salt extract, and 10 mM CaCl_2_) was added to the reaction vessel under constant stirring at 37 °C.

The pH-stat method was used to investigate and analyze the effects of the PPI-, SPI-, RBPI-, and WPI-stabilized nanoemulsions on the lipolysis rates of the lipid in the corn oil. When simulating the digestion stage of the small intestine, 0.25 M NaOH was continuously added using a 10 mL burette to maintain pH stability at 7.0. The amount of NaOH added during digestion was recorded (at 1, 3, 5, 10, 20, 60, 90, and 120 min), and the lipolysis rate was calculated using the following formula [24]:

(5) $$\mathrm{Lipolysis}\left(\%\right)=\frac{V_{NaOH}\times C_{NaOH}\times M_{oil}\times 100\%}{m_{oil}\times 2}$$

where *V_NaOH_* is the volume of added NaOH; *C_NaOH_* is the concentration of NaOH (0.25 M); *m_oil_* is the corn oil total weight present in the emulsions (g); and *M_oi_*_l_ is the average molecular weight of corn oil decomposed triglyceride, which was calculated as follows:

(6) $$M_{oil}=\frac{3\times 1000\times 56}{190}\;g/\mathrm{mol}$$

where 190 is the saponification value (SV) of corn oil and 56 is the molecular weight of potassium hydroxide (g/mol).

In order to correct the underestimation of the total free fatty acid liberation, the “*back-titration*” was used according to Stillhart et al. with slight modification [25]. After the small intestine digestion, the pH was immediately adjusted to 9 by 1 M NaOH to reach the complete free fatty acids (*FFA*) ionization, and the same procedure was also performed with blank digestion without emulsion. Then, the latter NaOH volume was subtracted from the total NaOH volume to obtain the value of *FFA (back-titration*). The correction factor was calculated as the following formula and used to correct the lipolysis rates result of oil digestion in small intestine:

(7) $$Correction\;Factor\;=\frac{FFA\;\left(direct\;titration\right)+FFA\;\left(back-titration\right)}{FFA\;\left(direct\;titration\right)}$$

where *FFA* (*direct titration*) is the amount of *FFA* titrated at pH 7.0 after the digestion period.

### 2.6. Nanostructure of the Emulsions

The nanostructure was evaluated by transmission electron microscopy (TEM; EM 902A, ZEISS, Oberkochen, Baden-Württemberg, Germany) operating at 80 kV. TEM samples were prepared by depositing nanosystems on a carbon-coated copper grid counterstained with 1% (*w/v*) phosphotungstic acid. The samples were air-dried for TEM measurement.

### 2.7. Bioaccessibility of β-Carotene

After digestion of emulsions in the simulated small intestine phase, the digestive fluid was centrifuged at 10,000× *g* for 45 min at 4 °C. Collecting the middle layer of micelles with a syringe carefully, they were filtered through a 0.22 mm filter, and chloroform was added to the solution to extract the β-carotene. This extraction was repeated until the sample was colorless, in order to ensure the full extraction of β-carotene, and the absorbance was measured at 450 nm after dilution to a suitable multiple. The β-carotene concentration was determined using the standard curve mentioned in Section 2.4; its bioaccessibility was estimated using the following formula:

(8) $$Bioaccessibility\;\left(\%\right)=\frac{C_{micelle}}{C_{initial\;emulsion}}\times 100\%$$

where *C_micelle_* and *C_initial emulsion_* represent the content of β-carotene in the micelle phase and the initial nanoemulsion, respectively.

### 2.8. Nanoemulsion Stability under Different Conditions

#### 2.8.1. Stability of Nanoemulsions under Centrifugation

Next, 0.5 mL nanoemulsions were diluted 10 times with deionized water. After centrifuging at 1750× *g* for 15 min, the 0.8 mL subnatant was withdrawn from the bottom of the PE tube. Then, the sample was vortex-mixed to ensure it was homogeneous and diluted 500-fold with phosphate buffer solutions (pH = 7.0). The absorbance was measured at 450 nm. The constant of centrifugal stability *Ke* values were calculated as follows [26]:

(9) $$Ke=\frac{|A_{0}-A|}{A_{0}}\times 100\%$$

where *A*_0_ and *A* represent the absorbances of the nanoemulsions before and after centrifugation, respectively.

#### 2.8.2. Stability of Nanoemulsions under Different pH

The mean particle size and ζ-potential were determined to characterize the stability of nanoemulsions under different pH conditions (in the range of 2.0–8.0); 0.1 M/2.0 M HCl/NaOH were used to adjust the pH value of the sample.

#### 2.8.3. Stability of Nanoemulsions under Different Storage Days

PPI-, SPI-, RBPI-, and WPI-stabilized nanoemulsions were stored for 28 days at 4 °C. The mean particle sizes were measured and the phenomena of nanoemulsions (e.g., the creaming layer) were also observed every 7 days in order to evaluate the nanoemulsions storage stability.

#### 2.8.4. Oxidation Stability of Nanoemulsions

The peroxide value (POV) of the nanoemulsions were measured as in Qiu et al. [27] with some modifications. Briefly, 0.2 mL of nanoemulsions was mixed with 1.5 mL of isooctane:isopropanol (3:1 *v/v*), vortexed, and centrifuged at 5000× *g* for 30 min. Then, 0.2 mL of upper layer was taken out and mixed with 2.8 mL of methanol:1-butanol (2:1 *v/v*). Then, the mixture was reacted with 15 μL of 3.94 M ammonium thiocyanate and 15 μL of ferrous solution (prepared by reacting 0.132 M cerium chloride with 0.144 M ferrous sulfate). After standing for 20 min, the absorbance of the mixture was measured at 510 nm. The hydroperoxide concentration was determined based on the standard curve of cymene hydroperoxide.

The method of thiobarbituric acid reactive substances (TBARS) measurement referred to that reported in [28]. Briefly, 1.0 mL of the PPI-, SPI-, RBPI-, and WPI-stabilized nanoemulsions were mixed with 2.0 mL of TBA (thiobarbituric acid) solution (prepared by mixing 15 g of trichloroacetic acid, 1.76 mL of 12 M HCl, 0.375 g of TBA, and 82.9 mL of H_2_O) in a tube and boiled for 15 min. Then, the cooled sample was centrifuged at 440× *g* for 15 min. The absorbance was measured at 532 nm. The concentration of TBARS was calculated from the standard curve of 1,1,3,3-tetraethoxypropane.

### 2.9. Statistical Analysis

All experiments were measured for three times. Results are expressed as mean ± standard deviation. The significance of the difference between the means was determined by Duncan’s multi-range test (*p* < 0.05) using the SPSS V20.0 (IBM Inc., New York, NY, USA, 2004).

## 3. Results and Discussion

### 3.1. Encapsulation Efficiency of β-Carotene

Encapsulation efficiency refers to the percentage of lipophilic nutrients loaded in the oil phase, which is an important indicator to evaluate the carrier performance of a nanoemulsion. As shown in Table 1, the encapsulation efficiencies of β-carotene in PPI-, SPI-, RBPI-, and WPI-stabilized nanoemulsions were all higher than 90%, with no significant difference (*p* > 0.05). Borba et al. [29] used a high-pressure homogenization method to stabilize a nanoemulsion and obtained over 98% encapsulation efficiency. Wei et al. [30] stabilized nanoparticles by emulsification evaporation and obtained nanoparticles with encapsulation efficiency up to 69%. It can be stated that the choice of nano-system and the method of preparation determine the difference in encapsulation efficiency. When the encapsulation efficiency was high in this study, it means that during the formation of the nanoemulsion, the oil phase encapsulated most of the β-carotene; that is, the protein emulsifiers could isolate the oil phase from the aqueous phase, wrap most of the β-carotene, and stabilize the β-carotene in the oil phase as a homogeneous dispersion.

### 3.2. Analysis of β-Carotene Nanoemulsion during In Vitro Digestion

#### 3.2.1. Oral Phase

**Figure 1:**
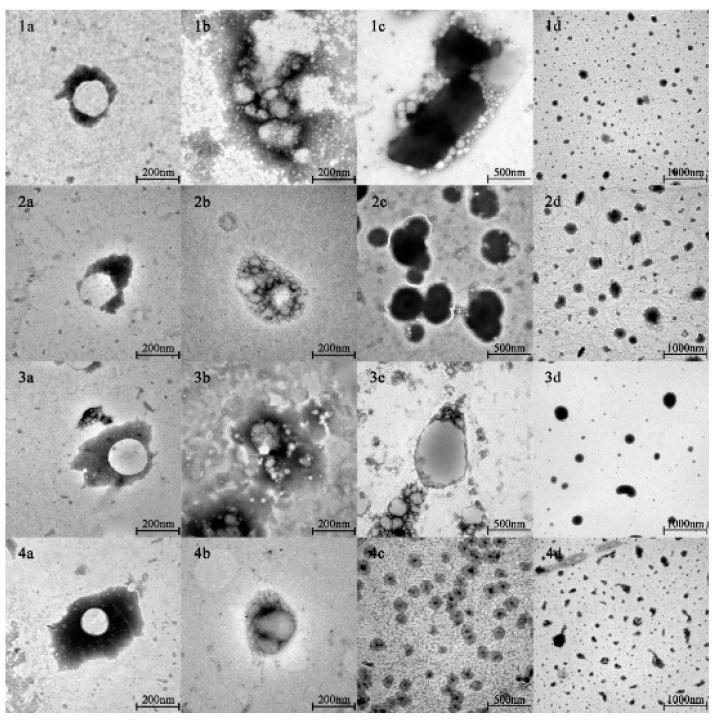
The nanostructures of PPI-, SPI-, RBPI-, and WPI-stabilized nanoemulsions in different digestion phases. (**1**–**4**) show the nanostructures of PPI-, SPI-, RBPI-, and WPI-stabilized nanoemulsions, respectively. (**a**–**d**) show the nanostructures of nanoemulsions in initial, oral, gastric, and small intestine phases, respectively.

**Figure 2:**
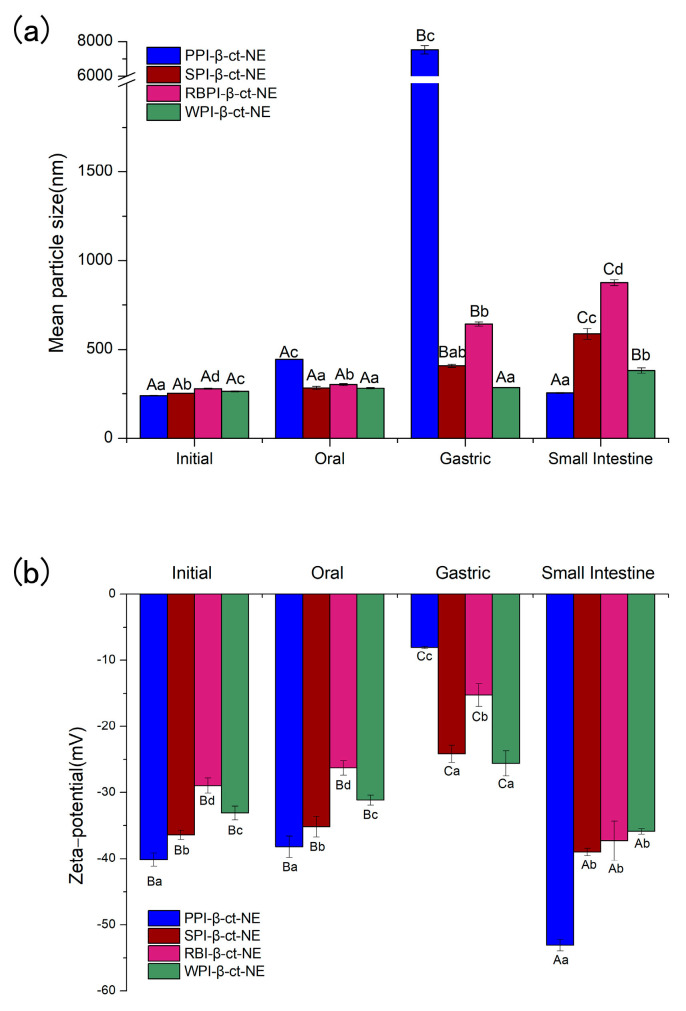
(**a**) The mean particle sizes of PPI-, SPI-, RBPI-, and WPI-stabilized nanoemulsions in different digestion phases; and (**b**) the zeta potential values of PPI-, SPI-, RBPI-, and WPI-stabilized nanoemulsions in different digestion phases. A–C represent that there were statistically significant differences (*p* < 0.05) in different digestion phases for the same sample. a–d represent that there were statistically significant differences (*p* < 0.05) for different samples in the same digestion phase.

The spherical droplets in nanoemulsions stabilized by PPI, SPI, RBPI, and WPI were irradiated by TEM, as shown in Figure 1(1a–4a). According to Table 1 and Figure 2 (specific data shown in Tables S1 and S2 of Supplementary Materials), it can be found that all emulsions had relatively small mean particle diameters and relatively small polydispersity indices (PDI < 0.2), which meant that the initial nanoemulsions were uniformly dispersed (the size distribution profiles of emulsions are shown in Supplementary Materials as Figure S2). In Figure 2b, it can be observed that the PPI-, SPI-, RBPI-, and WPI-stabilized nanoemulsions were negatively charged in the initial phase. This was because their pH was equal to 7.0, which is greater than the proteins isoelectric point (pI) value (about 4–5). Compared with the initial phase, the mean particle size in nanoemulsions was slightly increased after entering the oral digestion phase, but without significant differences, which is consistent with the dynamic light scattering data shown in Figure 2a. It also can be seen from Figure 1(1b–4b) that all nanoemulsions retained their nanostructures after the oral digestion phase. Previous research [31] showed that nanoemulsions may produce a small amount of bridging or depletion flocculation in simulated saliva, resulting in a significant increase in particle size. However, this phenomenon was not observed here. This indicates that the mucin in the simulated saliva was not sufficient to alter the emulsifier structure and cause extensive aggregation of the droplets in this work.

#### 3.2.2. Gastric Phase

As shown in Figure 1(1c–4c) and Figure 2a, after the gastric phase, the mean particle sizes of the PPI-, SPI-, and RBPI-stabilized nanoemulsions were highly increased and significantly different from their initial and oral phases (*p* < 0.05). On the contrary, there was no significant difference in mean particle size in the WPI-stabilized nanoemulsion. Firstly, the protein was hydrolyzed by pepsin, and the structure of the original emulsifier was disrupted, causing the droplets to aggregate in PPI-, SPI-, and RBPI-stabilized nanoemulsions. Secondly, after entering the gastric phase, the relatively low pH in the stomach made the negative droplets become more positive; thereby, the cationic lipid droplets and anionic mucin molecules could promote bridging, flocculation, and droplet aggregation [31]. Finally, the high ionic strength of the gastric fluid weakened electrostatic interactions, also favoring droplet aggregation, which caused a significant reduction (*p* < 0.05) in ζ-potential values (Figure 2b) [32,33].

In addition, it is worth noting that visible phase separation was seen during the gastric digestion of the PPI-stabilized nanoemulsion. Previous studies [34] have illuminated that the main components of PPI—arachin, conarachin I, and conarachin II—are all sensitive to proteases. Among them, conarachin I is sensitive to pepsin and trypsin. On the other hand, it is well known that β-sheet structures are stable in the process of gastric digestion due to their strong anti-protease digestion characteristics [35]. Therefore, the acidic environment stress of the emulsifier against the stomach phase may be stronger when the protein emulsifier possesses more β-sheet structures. As determined in our previous studies, the β-sheet structure of PPI is the lowest among PPI, SPI, RBPI, and WPI, which could have led to the ease of digestion of PPI [18]. According to the results of sodium dodecylsulfate polyacrylamide gel electrophoresis (SDS-PAGE) shown in Supplementary Materials as Figure S1, it can be found that most of the bands for PPI, SPI, and RBPI, especially PPI, disappeared, and only small peptides (<15 kDa) are visible, which suggested that the rapid degradation of PPI, SPI, and RBPI occurred. This result may also explain the phenomenon that PPI-stabilized emulsions had a more significant decrease in the zeta-potential values than the other emulsions, because the deep hydrolysis of PPI after gastric digestion tended to form large number of small peptides with lower or even zero charge, which could reduce the zeta potential values of emulsions more dramatically. Hence, we believe that the conformation damage of PPI was induced by deep hydrolysis, which was the main reason for its visible phase separation. On the contrary, the β-sheet structure of WPI was the highest among the four proteins and the majority of WPI remained intact after gastric digestion, which might explain the different digestion phenomena of the WPI-stabilized group [18]. Anti-pepsin digestion makes the WPI-stabilized nanoemulsion an ideal carrier to prevent the early release of nutrients in humans.

#### 3.2.3. Small Intestine Phase

As shown in Figure 1(1d–4d) and Figure 2a, after entering the small intestine phase, the mean particle size of the PPI-stabilized nanoemulsion decreased significantly compared to that in the gastric phase (*p* < 0.05). However, the WPI-, SPI-, and RBPI-stabilized nanoemulsions showed a significant increase in particle sizes compared to the gastric phase (*p* < 0.05). It has been previously found that in the small intestine digestion stage, bile salts can replace the interface molecules, resulting in competitive adsorption with the original emulsifier [36]. Consequently, the mean particle sizes of nanoemulsions in the small intestinal phase depended on the competitive adsorption result among bile acids, proteins, and small peptides [36]. As mentioned above (in Section 3.2.2), as PPI is sensitive to proteases, it tended to be deeply hydrolyzed in the stomach after 1 h of digestion, and plenty of PPI small peptides may be generated. PPI small peptides showed poor emulsifying capacity, which were more easily replaced by bile salts or lipolysis products, forming small bile salt micelles or fatty acid vesicles [37]. Hence, for the PPI-stabilized group with decreasing mean particle size, bile salts and fatty acid act as the important factor in forming mixed micelles. However, for WPI-, SPI-, and RBPI-stabilized groups, the mean particle size increased. This may because their structure was digested by trypsin to form small peptides with certain emulsifying capacity, which replaced the original protein to act as the main emulsifiers. As small peptides lacked sufficient surface tension to stabilize the emulsion, the droplet size then increased [38].

In the small intestine phase, the absolute value of ζ-potential for all groups increased significantly (*p* < 0.05; Figure 2b). This result could be attributed to the fact that bile salts, proteins, peptides, or lipolysis products could accumulate on the colloidal particles after lipid digestion, which resulted in negative charge growth [38]. In addition, the degree of ζ-potential increase was related to the displacement of peptides by bile salts. Chen et al. [36] reported that the more peptides were replaced by bile salts, the larger ζ-potential absolute values were observed after small intestine digestion. From Figure 2b, it can be seen that compared to the gastric phase, the absolute zeta-potential value of the PPI-emulsified nanoemulsion was the highest at the end of the small intestine phase, which was followed by RBPI-, SPI-, and WPI-emulsified groups. This phenomenon indicated the degree of peptide replacement was the highest for PPI [38]. Thus, our results provide more evidence to support that PPI peptides are easily substituted by bile salts or lipolysis products, thus achieving small mean particle size.

### 3.3. Lipid Digestion during In Vitro Digestion and Bioaccessibility of β-Carotene

**Figure 3:**
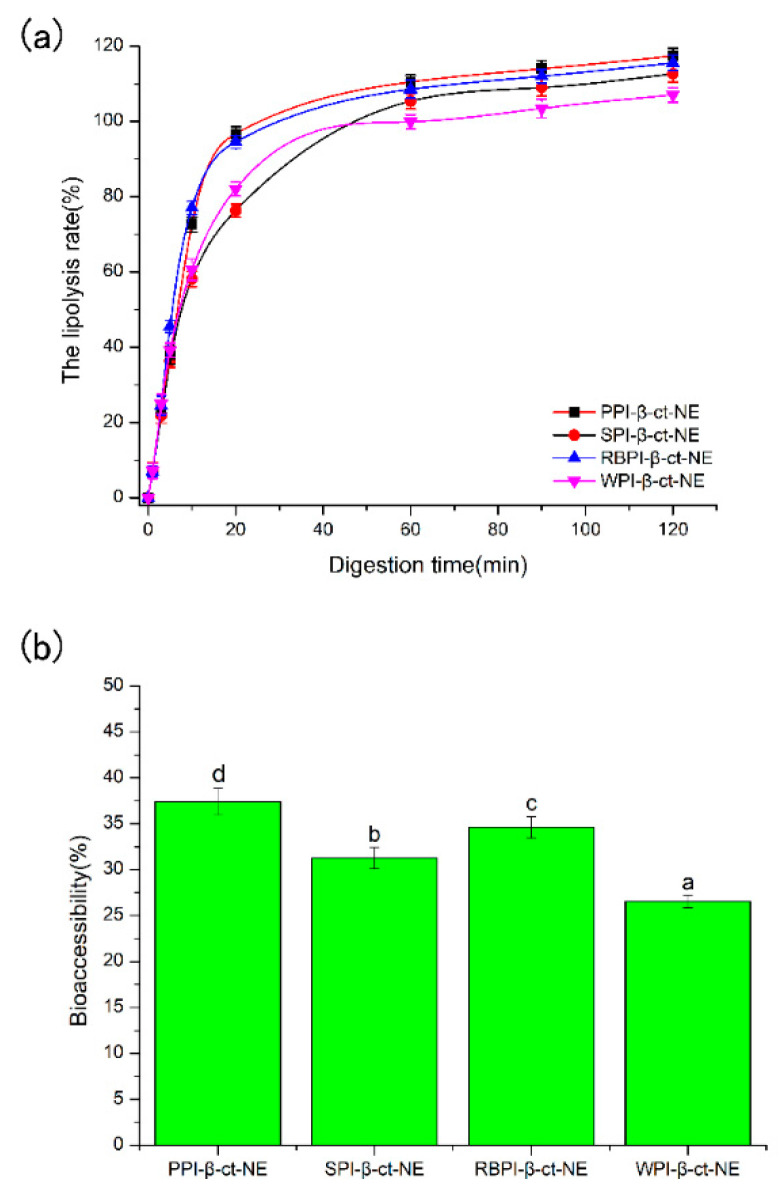
(**a**) The lipolysis rates of PPI-, SPI-, RBPI-, and WPI-stabilized nanoemulsions during the small intestine phase; and (**b**) the bioaccessibility of PPI-, SPI-, RBPI-, and WPI-stabilized nanoemulsions after in vitro digestion. a–d represent that there were statistically significant differences (*p* < 0.05) between PPI-, SPI-, RBPI-, and WPI-stabilized nanoemulsions, in terms of bioaccessibility. The lines are for visual aid only and the error bars are based on SD.

As shown in Figure 3a, the amount of *FFAs* increased rapidly in the first 30 min for all groups after entering the small intestine digestion phase. This suggests that lipase molecules rapidly adhered to the surfaces of the lipid droplets and promoted the digestion of the oil phase. Moreover, the protein emulsifier layer was partially replaced by bile salts and phospholipids, which benefitted lipase attachment and further accelerated the oil phase digestion. The amount of *FFAs* gradually increased with the extension of digestion time (30–120 min), and the rate of lipolysis finally reached a stable level. As seen from Figure 3a, the four nanoemulsions all achieved a high level of lipolysis rate. This reveals that the protein emulsifiers did not inhibit the proximity of lipase and reduce lipolysis. The PPI-emulsified group eventually reached a maximum lipolysis rate for about 117.39%. As bile salts dominated the competitive adsorption in the small intestine digestion stage for PPI-emulsified group, it can be deduced that the bile salt content in the mixed micelles of the PPI-emulsified group was greater than that in the other three groups. Hence, there may have been more active lipase sites at the mixed micelle interface [39]. In addition, the mean particle size of the PPI-emulsified group was significantly smaller than that of the other three groups, such that the larger droplet surface area might be exposed to more digestive enzymes, thus releasing more *FFA* [40]. Finally, glycerol and free fatty acids may be generated by the monoacylglycerol produced by lipolysis, which decreases the pH of the digestive product, such that the unconsidered requirement of NaOH increased the lipolysis rate [41]. This may be one reason causing lipolysis rates over 100%.

In comparison, the animal protein (WPI)-stabilized nanoemulsion obtained the lowest lipolysis among the four protein types. Studies by Li et al. [42] and Nik et al. [43] have both shown that the degree of lipolysis is closely related to the zeta-potential and particle size. Their experimental results showed that the lipolysis of SPI-stabilized nanoemulsion was higher than that of WPI-stabilized nanoemulsion. On one hand, during digestion in the small intestine, a higher absolute value of zeta potential can make the surfaces of the droplets more likely to adsorb bile salts. Therefore, more active sites of pancreatic lipase were produced on the surface, leading to an increase in free fatty acid release rate [44]. On the other hand, numerous in vitro studies have shown that the rate of lipid hydrolysis becomes higher with a decrease in the particle size of droplets. The reduction in droplet size led to an increase in specific surface area of droplets, which facilitated its contact with pancreatic lipase and bile salts [45,46]. According to our experimental results, the PPI-stabilized nanoemulsion had the smallest mean particle size and largest zeta potential (Figure 2) among all the four protein types, reaching the highest lipolysis rates during in vitro digestion.

The bioaccessibility of β-carotene was determined by measuring the amount of β-carotene in the mixed micelles at the end of digestion. After digestion and centrifugation, the emulsion samples were separated into three phases, with a thin oily or creamed phase, a clear micelle phase, and an opaque sediment phase at the top, middle, and bottom, respectively. It should be noticed that majority of the fatty acids/monoglycerides are located in the top creamed phase, which contains a large amount of β-carotene. Hence, the low bioaccessibility of β-carotene (<40%), compared to the high lipolysis rate (>100%), was obtained, because only the micelle phase was collected to calculate the bioaccessibility of β-carotene. These mixed micelles consisted of bile salts and phospholipids from intestinal fluids as well as free fatty acids and monoacylglycerols produced by lipid phase digestion. As shown in Figure 3b, the bioaccessibility of the PPI-stabilized group was the highest (37.39%), followed by the RBPI- (34.59%), SPI- (31.25%), and WPI-stabilized (26.54%) groups. The total amount of β-carotene released had a positive correlation with the final FFAs decomposition, which is consistent with previous studies [47]. Faster lipid digestion can improve the β-carotene released. Hence, the order of β-carotene bioaccessibility (PPI-NE > RBPI-NE > SPI-NE > WPI-NE) was consistent with the order of lipolysis rates (PPI-NE > RBPI-NE > SPI-NE > WPI-NE). Although the four biomacromolecular proteins were all suitable emulsifiers to prepare nanoemulsions with good bioaccessibility, the plant proteins PPI, RBPI, and SPI demonstrated better results than the animal protein WPI. Thus, PPI was definitely the optimal protein for nutrient loading, due to its highest lipolysis rate induced by small particle size and high bile salt substitution.

### 3.4. Nanoemulsion Stability Analysis under Different Conditions

#### 3.4.1. Stability of Nanoemulsions under Centrifugation

**Figure 4:**
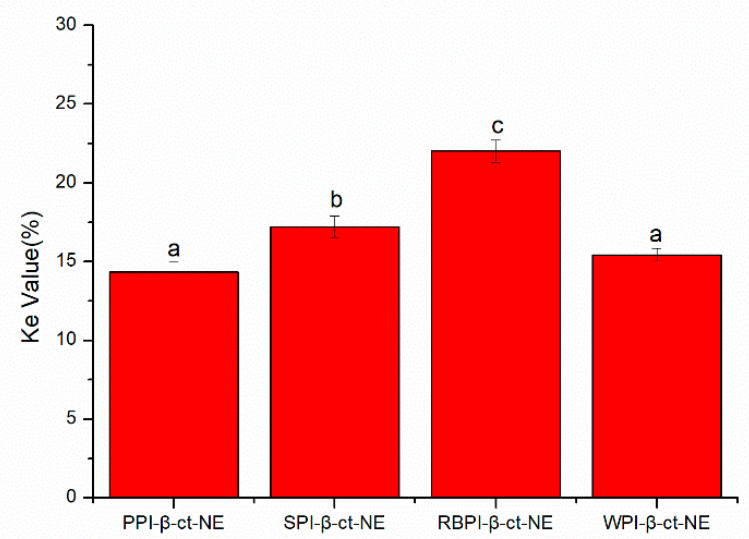
*Ke* values of PPI-, SPI-, RBPI-, and WPI-stabilized nanoemulsions. a–d represent statistically significant differences (*p* < 0.05) between PPI-, SPI-, RBPI-, and WPI-stabilized nanoemulsions, in terms of *Ke* values.

The centrifugal stability constant, *Ke*, represents the physical stability of the nanoemulsion. The smaller the *Ke* value, the higher the stability of the nanoemulsion. In Figure 4, the *Ke* values of the PPI-, SPI-, RBPI-, and WPI-stabilized nanoemulsions are all shown to be less than 30%, indicating that they were relatively stable. This was due to the large molecular weights and rigid molecular structures of the proteins, which could enhance the steric hindrance of the nanoemulsion to resist centrifugal force. Moreover, the variation trend of *Ke* values was basically the same as that of the mean particle sizes: PPI < WPI < SPI < RBPI. This confirmed that the droplet size was a key factor affecting the stability of the nanoemulsion. On the other hand, the absolute values of ζ-potentials in the PPI- and WPI- stabilized nanoemulsions were significantly higher than the other two at pH = 7. Therefore, the electrostatic repulsion force was also higher, leading to better centrifugal stability. The absolute value of the zeta potential was the lowest in the RBPI-stabilized group. Consequently, its electrostatic repulsion was weakest, and its centrifugal stability was poor, thus revealing that the zeta potential and electrostatic repulsion may be also important factors affecting *Ke*.

#### 3.4.2. Stability of Nanoemulsions under Different pH Values

**Figure 5:**
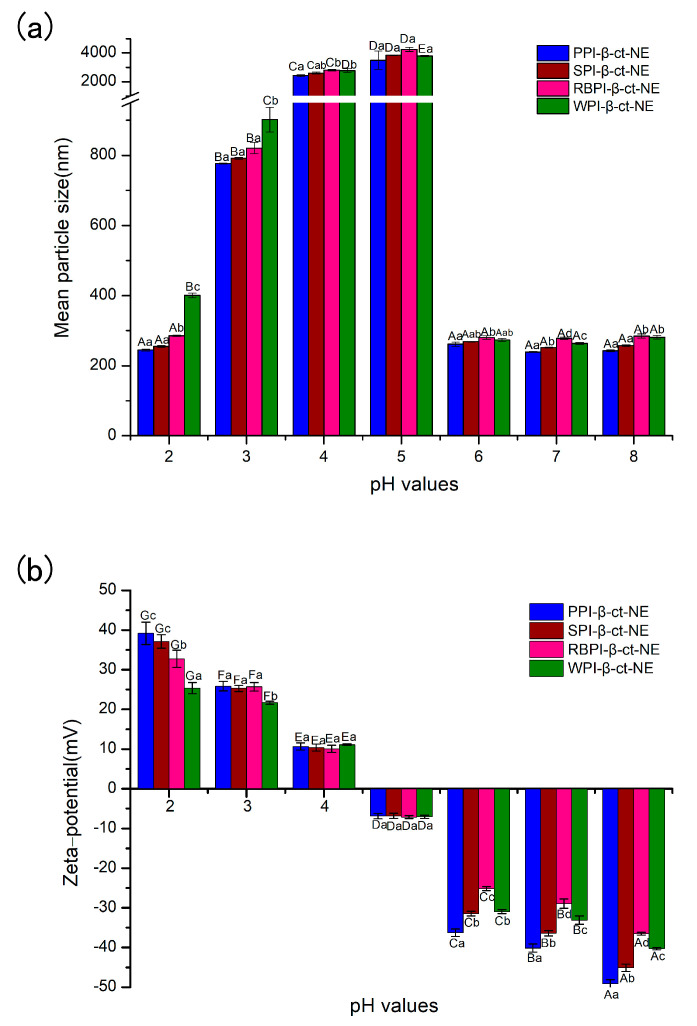
(**a**) Mean particle sizes of PPI-, SPI-, RBPI-, and WPI-stabilized nanoemulsions under different pH conditions; and (**b**) zeta potentials of the PPI-, SPI-, RBPI-, and WPI-stabilized nanoemulsions under different pH conditions. A–E represent statistically significant differences (*p* < 0.05) under different pH conditions in the same sample. a–d represent statistically significant differences (*p* < 0.05) between different samples under the same pH condition.

Nano-delivery systems are widely used in the food and pharmaceutical industries, especially in food processing, where some foods (e.g., soft drinks) may be processed under a range of pH environmental conditions. Therefore, it is essential to investigate the pH stability of nano-delivery systems. Herein, the mean particle sizes and zeta potentials of nanoemulsions at different pH conditions (from 2–8) were measured, in order to characterize their pH stability. In Figure 5a, the PPI-, SPI-, RBPI-, and WPI-stabilized groups are shown to be stable, where no visible phase separation was achieved over the whole pH range, except near the isoelectric point (pH = 4–5). This may be explained by the fact that the protein zeta-potential values were zero close to their own pI value. Obviously, electrostatic repulsion can maintain the homogeneous dispersion in nanoemulsions [48]. In the case of high positive charge and high negative charge, the adsorbed protein layer was able to prevent oil droplets from gathering. When the protein approached its pI, electrostatic repulsion was not sufficient to overcome Van der Waals forces and hydrophobic attraction, causing droplet aggregation. However, it was found that there was no significant color change, even though the nanoemulsion became highly aggregated at pI. This proves that β-carotene did not undergo any major physical or chemical changes due to the effects of pH, which is consistent with the results of previous research [49].

#### 3.4.3. Stability of Nanoemulsions under Different Storage Days

**Figure 6:**
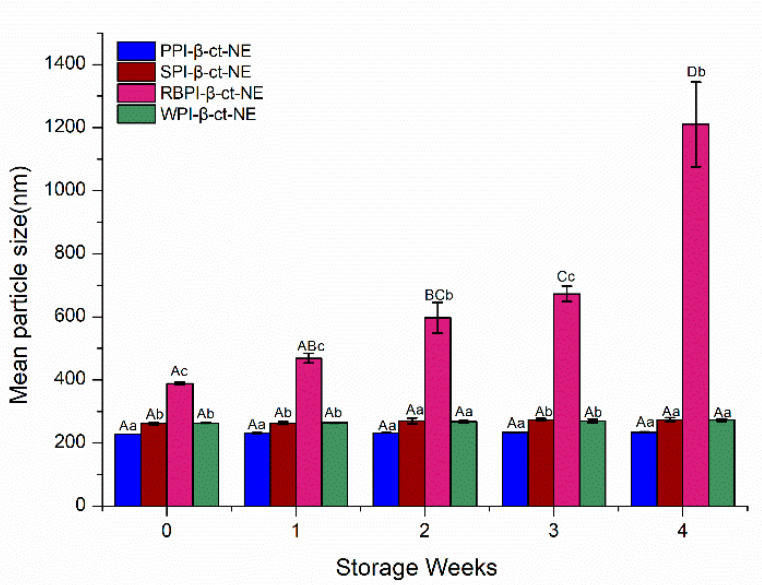
Mean particle sizes of the PPI-, SPI-, RBPI-, and WPI-stabilized nanoemulsions under different storage periods. A–D represent statistically significant differences (*p* < 0.05) in mean particle size under different storage weeks in the same sample. a–c represent statistically significant differences (*p* < 0.05) in mean particle size between different samples under the same storage week.

The storage stability of nanoemulsions is a vital factor in determining their suitability in the food industry. The main mechanisms of nanoemulsion instability include aggregation, coalescence, flocculation, and Ostwald ripening [50]. The storage stability of the PPI-, SPI-, RBPI-, and WPI-stabilized nanoemulsions were evaluated by measuring their particle size changes over 4 weeks. From Figure 6, it can be seen that the PPI-, SPI-, and WPI-stabilized groups remained stable for 4 weeks; their particle sizes did not change significantly (*p* > 0.05) and no creaming layer was observed during the storage time, which was very resistant to phase separation. This may have been due to the reason that the nanoemulsion droplets were fairly stable to gravity separation by forming a strong interface film with higher steric hindrance. However, the mean particle size of the RBPI-stabilized nanoemulsion was greater than 1000 nm over 4 weeks, and a creaming layer was observed during the storage time; that is, flocculation or coalescence had occurred, resulting in its poor physical stability during storage [51]. Thus, it can be concluded from the experiments above that PPI-, WPI-, and SPI-stabilized nanoemulsions showed appropriate stability during storage for 4 weeks storage.

#### 3.4.4. Oxidation Stability of Nanoemulsions

**Figure 7:**
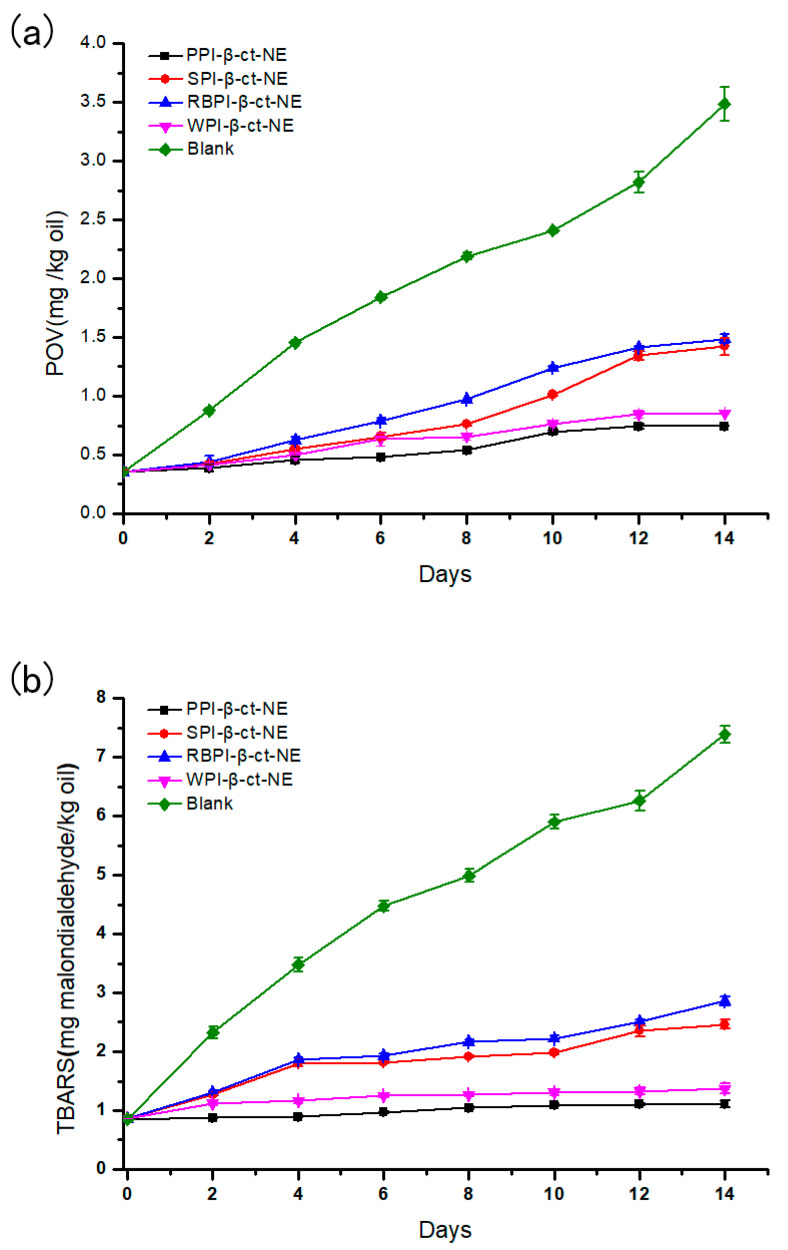
(**a**) Peroxide values (POVs) of PPI-, SPI-, RBPI-, and WPI-stabilized nanoemulsions over time; and (**b**) thiobarbituric acid reactive substances (TBARS) values of PPI-, SPI-, RBPI-, and WPI-stabilized nanoemulsions over time. The lines are for visual aid only and that the error bars are based on SD.

The PPI-, SPI-, RBPI-, and WPI-stabilized nanoemulsions were stored at 4 °C for 14 days to evaluate their primary oxidation (POV) and secondary oxidation (TBARS) stabilities, while the same oil content was used as a blank control group. As shown in Figure 7 (specific data shown in Tables S3 and S4 of Supplementary Materials), the two oxidation rates of the four nanoemulsions were lower than those of the control group, thus proving that the nanoemulsions could effectively reduce oxidation [52]. In the initial stage of storage, all groups showed an increase in both POV and TBARS values. This indicated that some degree of lipid oxidation had occurred. For the primary lipid hydroperoxide over 14 days of storage, the oxidation rates of the PPI- and WPI-stabilized groups were lower than those of the SPI- and RBPI-stabilized groups. Moreover, the rate of the PPI-stabilized group in producing secondary oxidation products was also slower than the other three groups. Considering the two oxidation products, the PPI-stabilized nanoemulsion was the slowest in the process of lipid oxidation and had the best oxidative stability of the four nanoemulsions. The WPI-stabilized nanoemulsion had the second-best oxidation stability, which was followed by the SPI- and RBPI-stabilized groups, respectively.

Due to the presence of the emulsifier layer, the oil phase did not make direct contact with the air or water. Therefore, oxidation mainly occurred at the interfaces of oil droplets. Thus, it was realized that the selection of emulsifier affects the oxidation stability of the nanoemulsion powerfully. Jacobsen et al. [53] mentioned that the oxidation stability of a nanoemulsion can be attributed to the thickness of the interface, the charge carried by the interface, and the ability of the emulsifier to bond with the metal. It may be considered that the higher absolute ζ-potentials of PPI and WPI favored the combination between protein and pro-oxidants in the aqueous phase, leading to the stronger oxidation stability of the associated nanoemulsions. In contrast to previous studies, in our investigation, the RBPI-stabilized group was more likely to form a small range of droplet aggregation due to its poor storage stability, together with some extent of oxidation in the oil phase [8]. However, the RBPI oxidation stability was not extremely worse than that of the other three groups, as shown in Figure 7. The antioxidant properties of nanoemulsions are affected by many factors, while many potential mechanisms also affect the lipid oxidation of nanoemulsions.

## 4. Conclusions

In this study, we showed that both plant protein (PPI, SPI, and RBPI)-stabilized nanoemulsions and an animal protein (WPI)-stabilized nanoemulsion could effectively load β-carotene with a high encapsulation efficiency (90%) and present great nanoemulsion stability against centrifugation, various pH values, storage, and oxidation conditions. However, plant protein nanoemulsions, especially the PPI-stabilized nanoemulsion, showed better lipolysis rates and β-carotene bioaccessibility than the animal protein (WPI)-stabilized nanoemulsion. This result may be related to the smaller particle size of the PPI-stabilized nanoemulsion after small intestine digestion, which exposed the larger droplet surfaces to digestive enzymes, thus releasing more *FFAs*. Furthermore, the high bile salt or substitution of PPI-stabilized nanoemulsion may also provide more active lipase sites at the mixed micelle interface to promote the oil digestion and increase the β-carotene bioaccessibility. The PPI-stabilized nanoemulsion performed relatively well and showed outstanding stability compared to WPI-, SPI-, and RBPI-stabilized nanoemulsions, based on the *Ke* value, pH, storage, and oxidation stability results. Therefore, the results of this study can provide more information about the development of the plant protein emulsifiers, which are low cost and abundant resources, and are beneficial to the complete utilization of plant proteins in food industry. However, it is worth noting that due to the complexity of the food system in production practice, research, and analysis of nanoemulsions, more complex and realistic digestive and storage systems should be considered in further research.

## Figures and Tables

**Table 1:** The encapsulation efficiency (EE) of β-carotene and polydispersity indices (PDI) in different digestion stages for the peanut protein isolate (PPI)-, soy protein isolate (SPI)-, rice bran protein isolate (RBPI)-, and whey protein isolate (WPI)-stabilized nanoemulsions (PPI-β-ct-NE, SPI-β-ct-NE, RBPI-β-ct-NE, WPI-β-ct-NE).

	EE%	PDI (Polydispersity Index)			
		Initial	Oral	Gastric	Small Intestine
PPI-β-ct-NE	92.23 ± 0.95 ^a^	0.087 ± 0.008 ^Aa^	0.274 ± 0.003 ^Bc^	0.812 ± 0.060 ^Cc^	0.135 ± 0.005 ^Aa^
SPI-β-ct-NE	90.87 ± 2.57 ^a^	0.094 ± 0.006 ^Aa^	0.098 ± 0.007 ^Aa^	0.291 ± 0.022 ^Ba^	0.262 ± 0.032 ^Bb^
RBPI-β-ct-NE	93.81 ± 0.55 ^a^	0.181 ± 0.044 ^Ab^	0.217 ± 0.007 ^Ab^	0.276 ± 0.017 ^Ba^	0.353 ± 0.015 ^Cc^
WPI-β-ct-NE	92.13 ± 0.96 ^a^	0.143 ± 0.005 ^Ab^	0.205 ± 0.009 ^Bb^	0.467 ± 0.028 ^Cb^	0.522 ± 0.014 ^Dd^

^A–D^ indicate that there were statistically significant differences (*p* < 0.05) in different digestion phases for the PDI of same sample. ^a–d^ indicate that there were statistically significant differences (*p* < 0.05) in the EE% and the PDI of different samples in the same digestion phase.

## Data Availability

Data presented in this study are available by requesting from the corresponding author.

## Supplementary Materials

The following are available online at https://www.mdpi.com/2079-4991/11/1/167/s1, Figure S1: Sodium dodecylsulfate polyacrylamide gel electrophoresis (SDS-PAGE) of PPI, SPI, RBPI and WPI after gastric digestion, Figure S2: The PDI of PPI-, SPI-, RBPI-, and WPI-stabilized nanoemulsions in different digestion phases, Table S1: The mean particle size (nm) in different digestion stage of the PPI-, SPI-, RBPI-, and WPI-stabilized nanoemulsions, Table S2: The zeta-potential (mV) in different digestion stage of the PPI-, SPI-, RBPI-, and WPI-stabilized nanoemulsions, Table S3: The POV values (mg/kg oil) in different days of the PPI-, SPI-, RBPI-, and WPI-stabilized nanoemulsions, Table S4: The TBARS values (mg malondialdehyde/kg oil) in different days of the PPI-, SPI-, RBPI-, and WPI-stabilized nanoemulsions.
