# Supplementary material for: Comparison of Different Protein Emulsifiers on Physicochemical Properties of β-Carotene-Loaded Nanoemulsion: Effect on Formation, Stability, and In Vitro Digestion
Source: Nanomaterials (Basel). 2021 Jan 11;11(1):167. doi: 10.3390/nano11010167 (PMC7826833; doi:10.3390/nano11010167)
Supplement: Supplementary file 1 [file nanomaterials-11-00167-s001.pdf]

nanomaterials

Supplementary Materials

Comparison of Different Protein Emulsifiers on Physicochemical Properties of β-Carotene-Loaded Nanoemulsion: Effect on Formation, Stability, and In Vitro Digestion

Yanlong Liu [1,†], Chang Liu [1,†], Shenyi Zhang [1], Jishu Li [1], Huanyu Zheng [2,3,4], Hua Jin [1,*] and Jing Xu [1,*]

1 College of Art and Science, Northeast Agricultural University, Harbin, Heilongjiang 150030, China; liuyanlonghyy@163.com (Y.L.); lovekursaal@163.com (C.L.); zsylq123@163.com (S.Z.); ljswy123@163.com (J.L.)
2 College of Food Science, Northeast Agricultural University, Harbin, Heilongjiang 150030, China; zhenghuanyu1@163.com
3 Heilongjiang Green Food Science Research Institute, Harbin, Heilongjiang 150028, China
4 National Research Center of Soybean Engineering and Technology, Harbin, Heilongjiang 150028, China
* Correspondence: jinhua@neau.edu.cn (H.J.); xujing@neau.edu.cn (J.X.); Tel.: +86-1874-571-7574 (H.J.); +86-1379-665-2155 (J.X.)
† These authors contributed equally to this work and should be considered co-first authors.

Table S1. The mean particle size (nm) in different digestion stage of the PPI-, SPI-, RBPI-, and WPI-stabilized nanoemulsions.

	PPI-β-ct-NE	SPI-β-ct-NE	RBPI-β-ct-NE	WPI-β-ct-NE
Initial	238.95 ± 1.77	251.40 ± 1.13	278.40 ± 3.25	263.70±3.11
Oral	444.45 ± 0.64	282.40 ± 9.48	301.45 ± 5.87	281.35 ± 3.61
Gastric	7528.5 ± 246.78	409.05 ± 8.41	642.25 ± 12.09	284.45 ± 1.06
Small Intestine	254.8 ± 2.69	587.5 ± 30.12	876.45 ± 16.33	381.6 ± 15.41

Table S2. The zeta-potential (mV) in different digestion stage of the PPI-, SPI-, RBPI-, and WPI-stabilized nanoemulsions.

	PPI-β-ct-NE	SPI-β-ct-NE	RBPI-β-ct-NE	WPI-β-ct-NE
Initial	−40.13 ± 1.02	−36.40 ± 0.69	−28.93 ± 1.16	−33.10 ± 1.04
Oral	−38.20 ± 1.69	−35.17 ± 1.55	−26.27 ± 1.10	−31.17 ± 0.74
Gastric	−8.04 ± 0.15	−24.13 ± 1.30	−15.23 ± 1.72	−25.57 ± 1.89
Small Intestine	−53.13 ± 0.85	−38.97 ± 0.55	−37.30 ± 2.98	−35.87 ± 0.42

Table S3. The POV values (mg/kg oil) in different days of the PPI-, SPI-, RBPI-, and WPI-stabilized nanoemulsions.

POV	PPI-β-ct-NE	SPI-β-ct-NE	RBPI-β-ct-NE	WPI-β-ct-NE	Blank
Day 0	0.358 ± 0.005	0.356 ± 0.005	0.352 ± 0.002	0.356 ± 0.004	0.355 ± 0.004
Day 2	0.388 ± 0.033	0.423 ± 0.016	0.443 ± 0.049	0.413 ± 0.019	0.877 ± 0.025
Day 4	0.456 ± 0.028	0.551 ± 0.015	0.623 ± 0.031	0.498 ± 0.023	1.454 ± 0.019
Day 6	0.479 ± 0.008	0.655 ± 0.030	0.789 ± 0.021	0.632 ± 0.055	1.843 ± 0.019
Day 8	0.540 ± 0.020	0.760 ± 0.013	0.974 ± 0.005	0.657 ± 0.033	2.189 ± 0.029
Day 10	0.696 ± 0.017	1.012 ± 0.016	1.241 ± 0.009	0.764 ± 0.011	2.412 ± 0.004
Day 12	0.742 ± 0.021	1.344 ± 0.039	1.412 ± 0.011	0.846 ± 0.032	2.820 ± 0.088
Day 14	0.745 ± 0.004	1.422 ± 0.070	1.487 ± 0.040	0.854 ± 0.010	3.488 ± 0.144

Table S4. The TBARS values (mg malondialdehyde/kg oil) in different days of the PPI-, SPI-, RBPI-, and WPI-stabilized nanoemulsions.

TBARS	PPI-β-ct-NE	SPI-β-ct-NE	RBPI-β-ct-NE	WPI-β-ct-NE	Blank
Day 0	0.851 ± 0.020	0.865 ± 0.016	0.859 ± 0.012	0.862 ± 0.013	0.854 ± 0.020
Day 2	0.877 ± 0.010	1.272 ± 0.015	1.306 ± 0.025	1.125 ± 0.046	2.327 ± 0.099
Day 4	0.896 ± 0.015	1.794 ± 0.047	1.868 ± 0.029	1.166 ± 0.040	3.481 ± 0.121
Day 6	0.972 ± 0.017	1.817 ± 0.021	1.932 ± 0.031	1.249 ± 0.027	4.474 ± 0.081
Day 8	1.045 ± 0.025	1.918 ± 0.015	2.167 ± 0.033	1.265 ± 0.033	4.991 ± 0.116
Day 10	1.092 ± 0.029	1.979 ± 0.037	2.224 ± 0.044	1.309 ± 0.049	5.904 ± 0.124
Day 12	1.105 ± 0.017	2.351 ± 0.093	2.506 ± 0.030	1.327 ± 0.061	6.260 ± 0.166
Day 14	1.109 ± 0.061	2.461 ± 0.079	2.859 ± 0.079	1.370 ± 0.082	7.389 ± 0.141

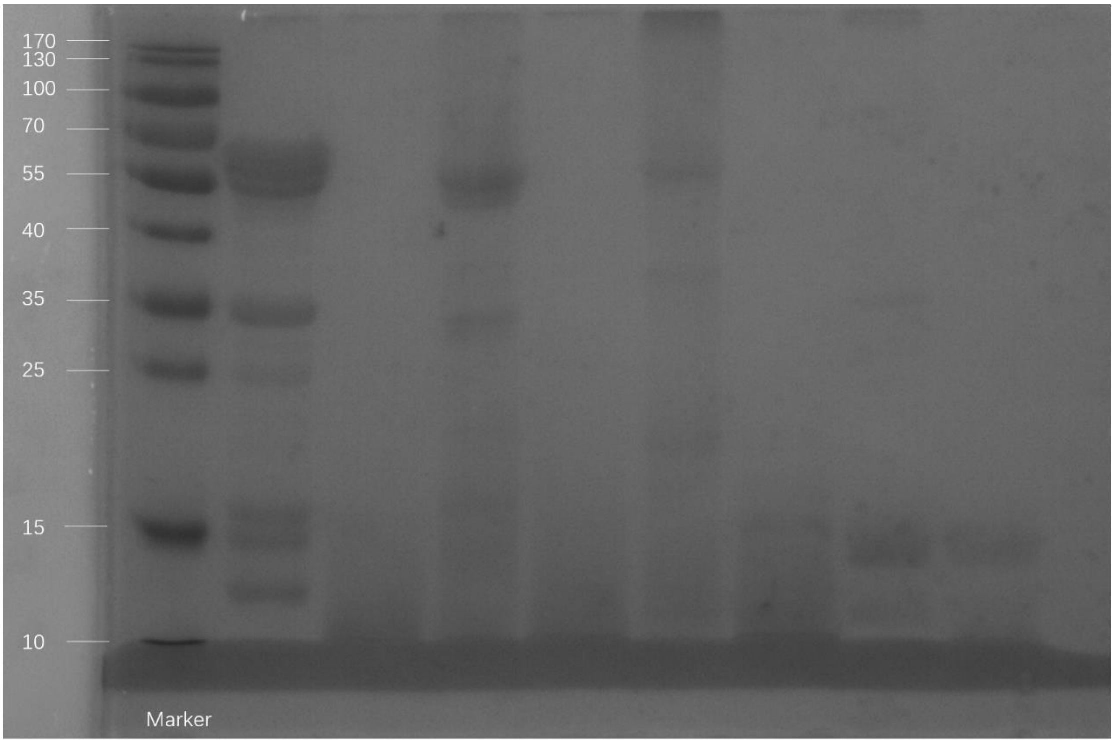

Figure S1. Sodium dodecylsulfate polyacrylamide gel electrophoresis (SDS-PAGE) of PPI, SPI, RBPI and WPI after gastric digestionFrom left to right, lane 1 and 2 are initial PPI sample and PPI sample after gastric digestion; lane 3 and 4 are initial SPI sample and SPI sample after gastric digestion; lane 5 and 6 are initial RBPI sample and BPI sample after gastric digestion; lane 7 and 8 are initial WPI sample and WPI sample after gastric digestion.

(1a)

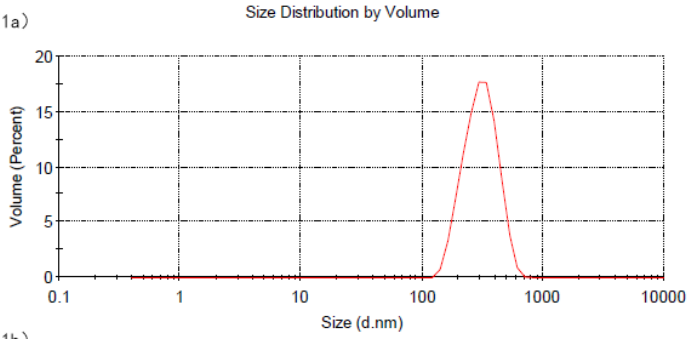

Size Distribution by Volume
Volume (Percent)
20
15
10
5
0
0.1
1
10
100
1000
10000
Size (d.nm)

(1b)

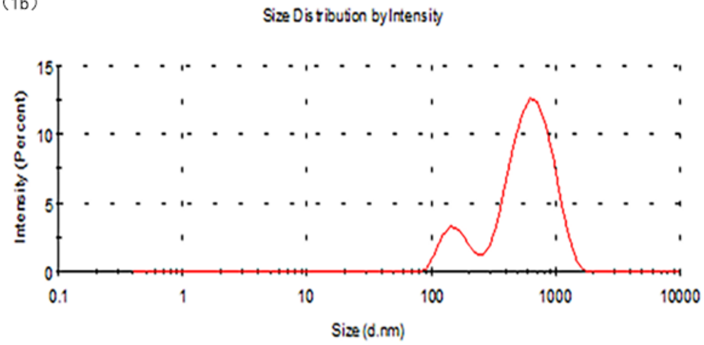

Size Distribution by Intensity
Intensity (Percent)
15
10
5
0
0.1
1
10
100
1000
10000
Size (d.nm)

(1c)

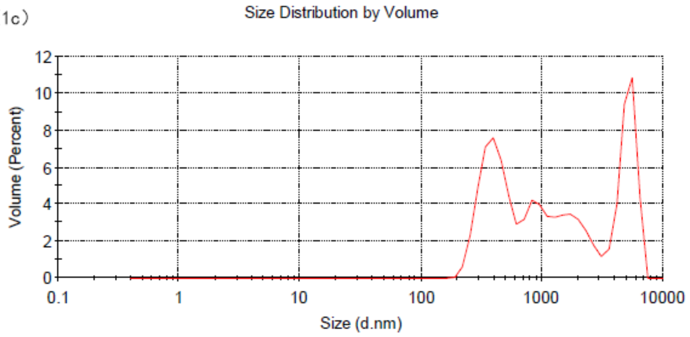

Size Distribution by Volume
Volume (Percent)
12
10
8
6
4
2
0
0.1
1
10
100
1000
10000
Size (d.nm)

(1d)

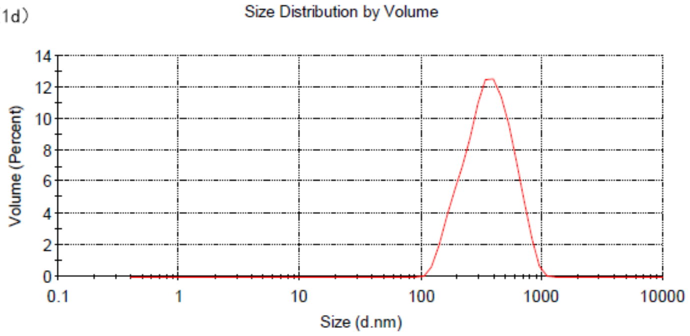

Size Distribution by Volume
Volume (Percent)
14
12
10
8
6
4
2
0
0.1
1
10
100
1000
10000
Size (d.nm)

(2a)

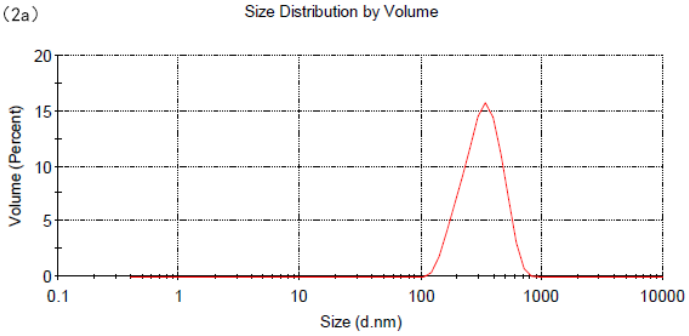

Size Distribution by Volume
Volume (Percent)
0
5
10
15
20
0.1
1
10
100
1000
10000
Size (d.nm)

(2b)

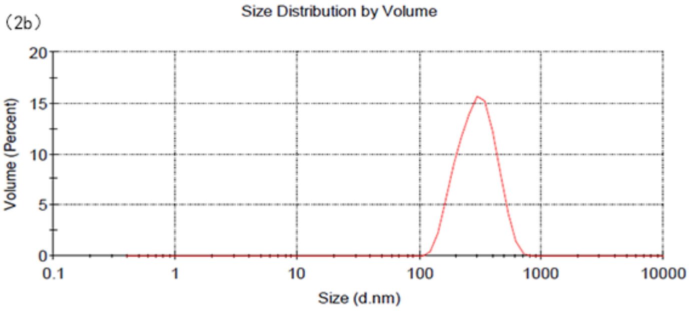

Size Distribution by Volume
Volume (Percent)
0
5
10
15
20
0.1
1
10
100
1000
10000
Size (d.nm)

(2c)

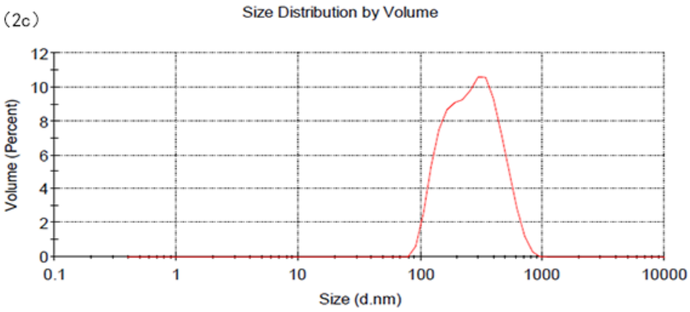

Size Distribution by Volume
Volume (Percent)
0
2
4
6
8
10
12
0.1
1
10
100
1000
10000
Size (d.nm)

(2d)

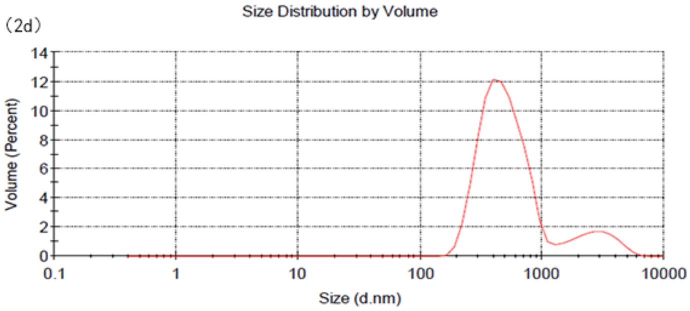

Size Distribution by Volume
Volume (Percent)
0
2
4
6
8
10
12
14
0.1
1
10
100
1000
10000
Size (d.nm)

(3a)

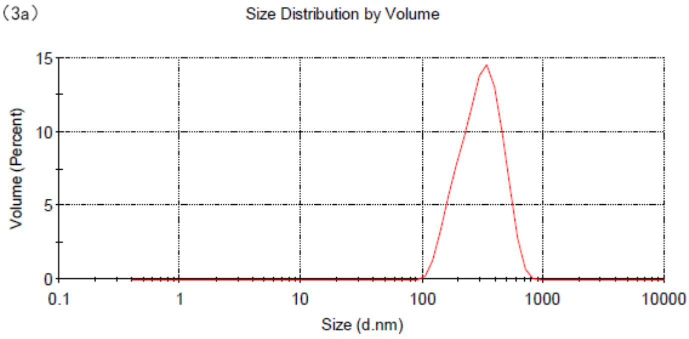
Size Distribution by Volume
Volume (Percent)
15
10
5
0
0.1
1
10
100
1000
10000
Size (d.nm)

(3b)

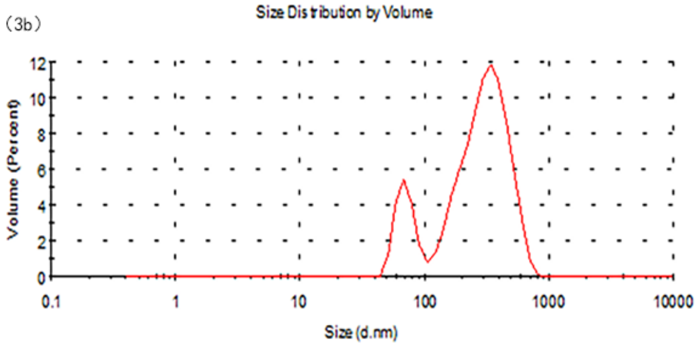
Size Distribution by Volume
Volume (Percent)
12
10
8
6
4
2
0
0.1
1
10
100
1000
10000
Size (d.nm)

(3c)

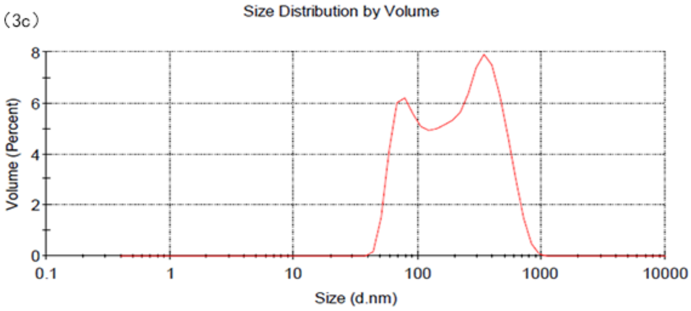
Size Distribution by Volume
Volume (Percent)
8
6
4
2
0
0.1
1
10
100
1000
10000
Size (d.nm)

(3d)

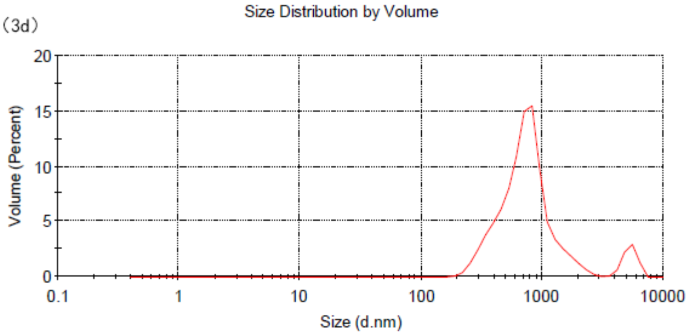
Size Distribution by Volume
Volume (Percent)
20
15
10
5
0
0.1
1
10
100
1000
10000
Size (d.nm)

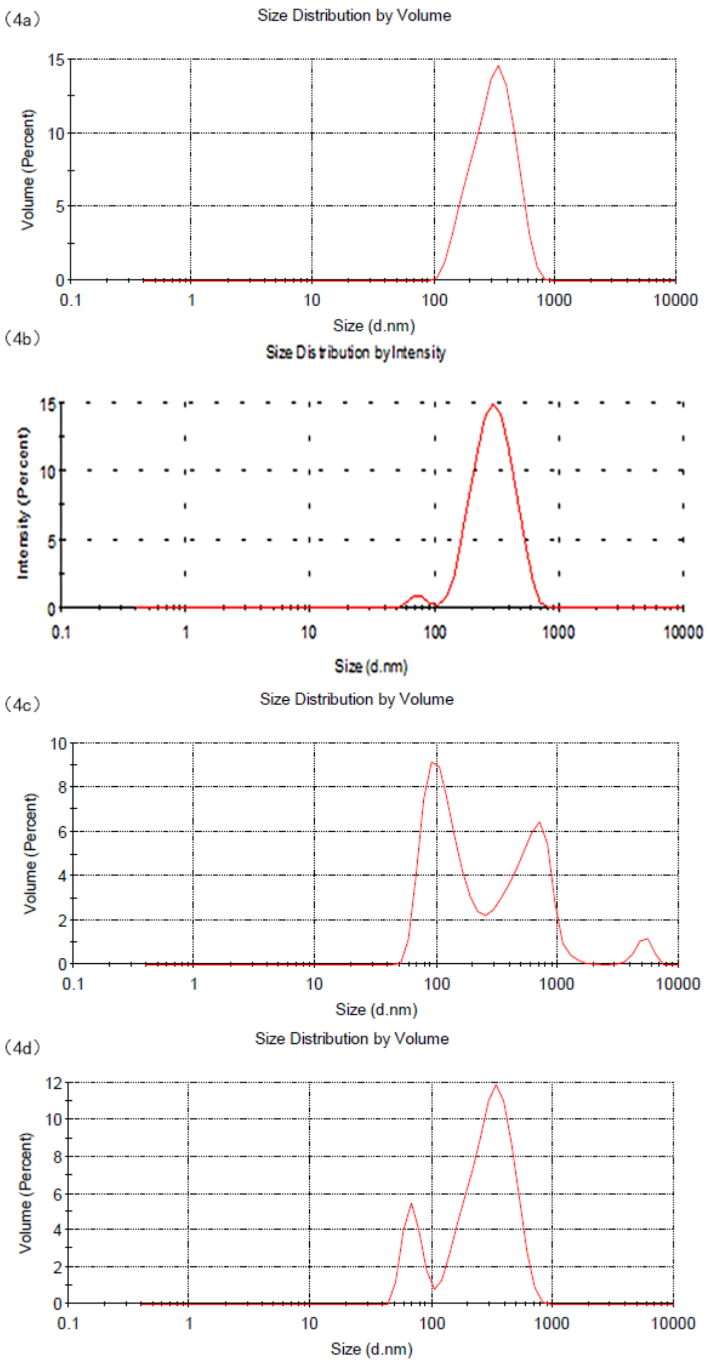

Figure S2. The PDI of PPI-, SPI-, RBPI-, and WPI-stabilized nanoemulsions in different digestion phases. **1–4** show the nanostructures of PPI-, SPI-, RBPI- and WPI-stabilized nanoemulsions, respectively. **a–d** show the nanostructures of nanoemulsions in initial, oral, gastric, and small intestine phases, respectively.
